# l‑Lactate Oxidase-Based Biosensor Enables Quasi-Calibration-Free Detection of l‑Lactate in Sweat of Acidic to Neutral pH

**DOI:** 10.1021/acssensors.5c01238

**Published:** 2025-06-06

**Authors:** Kosuke Ike, Kousuke Muto, Takahiro Hioki, Noya Loew, Isao Shitanda, Masafumi Takesue, Mitsuyoshi Okuda

**Affiliations:** † Laboratory of Biological & Material Science Research, 74004 Kao Corporation, 1334 minato, 640-8580 Wakayama, Japan; ‡ Laboratory of Performance Chemicals Research, Kao Corporation, 1334 minato, 640-8580 Wakayama, Japan; § Department of Pure and Applied Chemistry, Faculty of Science and Technology, 13258 Tokyo University of Science, 2641 Yamazaki, Noda 278-8510, Chiba, Japan; ∥ Laboratory of Biological & Material Science Research, 46618 Kao Corporation, 2606 Akabane, Ichikai, Haga 321-3497, Tochigi, Japan

**Keywords:** lactate biosensor, lactate oxidase, enzyme electrode, screen printing, l-lactate, human sweat, acid-tolerant

## Abstract

l-lactate biosensing has attracted attention in recent years in sports, medicine, and nursing care fields as well as in food manufacturing and biotechnology industries. In particular, l-lactate in human sweat, a biological indicator that can be collected noninvasively, has driven rapid progress in the research and development of sensing technology, positioning sweat as a new target to replace blood and interstitial fluid. The key to l-lactate sensing in human sweat, which contains various biological components, is using l-lactate oxidase (LOX) as a recognition element. l-lactate can be specifically, continuously, and quantitatively measured using this enzyme electrode. However, as conventional LOX is affected by acidic pH, biosensors must be calibrated for each individual for accurate l-lactate quantification owing to individual differences in sweat pH. Furthermore, fluctuations in sweat pH during exercise lead to inaccuracies in the detected l-lactate levels. Therefore, identifying LOX active in acidic pH is crucial. Here, we report a novel LOX with acidic pH tolerance and a technology that enables constant detection of l-lactate levels in acidic to neutral pH sweat. Phylogenetic analysis of α-hydroxy acid oxidase in a public protein database, with the evaluation of heterologously expressed enzymes, revealed the existence of a novel LOX with better acidic pH tolerance compared to that observed with conventional LOX. Furthermore, applying the novel LOX to a paper electrode screen-printed with MgO-templated carbon enhanced the l-lactate response at acidic pH compared to that observed with conventional enzyme electrodes while maintaining a pH-independent response to l-lactate. Overall, biosensors utilizing this novel LOX will be quasi-calibration-free, by eliminating the need for adjusting the calibration according to changes in pH. Thus, our findings contribute to expanding the use of l-lactate biosensors targeting sweat and accelerating their societal application.

## Graphical abstract

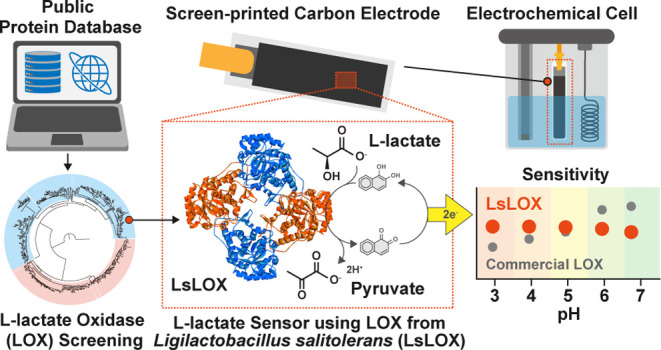

Recently, l-lactate biosensing technology has attracted increasing attention in sports, medicine, and nursing care fields as well as in food manufacturing and biotechnology industries.^1^ For example, in sports, l-lactate level serves as an important indicator for assessing the metabolic responses of the body, providing valuable data for developing training and recovery strategies for athletes.^2^ Commonly, l-lactate is measured in blood, which requires invasive sampling and is limited in terms of real-time measurement, continuity, and hygiene. Therefore, in recent years, the focus has shifted to l-lactate measurements in alternate biofluids, especially sweat.^2–7^

Researchers have taken various approaches to improve sweat l-lactate sensing devices. These devices have been optimized in terms of wearability, comfort, flexibility, sweat collection, etc. Various l-lactate-sensing devices have been developed, including patch-,^8^ fabric-,^9^ tattoo-,^10^ and paper-type devices.^11^ Furthermore, printing techniques are often utilized for the fabrication of these sensors.^6,9^ Both the resulting flexibility in the design, and the easy adaptability to mass production are desirable features of printing techniques.

One approach to increasing the response current of enzyme electrodes is to utilize electrode materials with a high specific surface area.^12^ One such mesoporous electrode material of interest is MgO-templated carbon (MgOC).^6,13^ MgOC is especially interesting as its pore size can be controlled by the MgO templates.^14,15^ Screen-printed MgOC electrodes have been used for a variety of biosensors and biofuel cells, including for the measurement of l-lactate in sweat.^6,16,17^

One major, yet often overlooked, challenge is that enzymatic l-lactate sensors loose sensitivity in acidic samples.^5,6,18,19^ This is significant because human sweat, though usually neutral, can be as acidic as approximately pH 4.0.^20,21^ Human sweat pH depends on the individual’s diet, chronic and acute health, and/or exercise conditions.^20,21^ A common approach to eliminate the influence of pH variation is the simultaneous measurement of l-lactate and pH;^6,22,23^ however, this requires multiple calibrations at different pH values as well as a decision mechanism of which calibration to use depending on the measured pH. A pH-independent l-lactate sensor would be preferable, and approaches to achieve this include the addition of a pH control layer,^24^ a flux restricting layer,^5^ or enzymatic encapsulation with hydrophobic carbon.^25^

The main reason for pH sensitivity is the activity of the enzyme used as recognition element, commonly l-lactate oxidase (EC 1.1.3.2, LOX). LOXs evaluated in previous studies, such as LOX from Aerococcus viridans (AvLOX),^26^ LOX from Enterococcus faecium (EfLOX),^27^ and commercially available LOX (e.g., LCO-301 (TOYOBO)), have optimal activity at neutral pH and weak activity under acidic conditions. Therefore, LOX sensing sensitivity is greatly reduced under acidic conditions.^24^ Despite the significant need for pH-independent l-lactate sensing, only a few studies have focused on improving the enzyme itself.

Here, we focused on improving LOX function and searched for novel acid-tolerant LOXs in a public protein database. We identified a novel LOX (LsLOX; LOX from Ligilactobacillus salitolerans) with improved acid tolerance compared to that observed with conventional LOX. Furthermore, we confirmed that applying this enzyme to an electrode enhanced l-lactate response at acidic pH as compared to that observed with the conventional enzyme electrode. Surprisingly, the pH-dependency of l-lactate sensing was also greatly suppressed.

## Materials and Methods

### Phylogenetic Analysis of α-Hydroxy Acid Oxidase

Reference sequences^28^ included those for mandelate dehydrogenase (PDB 1p4c), lactate monooxygenase (WP_096312069.1), glycolate oxidase (PDB 1GOX), cytochrome *b2* oxidase (PDB 1fcb), and five types of lactate oxidases (WP_003142047.1,^26^ WP_010723216.1,^27^ WP_024862288.1,^29^ NP_114471.1,^28^ and WP_236973973.1 ^30^). Lactate dehydrogenase from Homo sapiens (NP_001302466.1) was used as an outer group. Five thousand homologous sequences for each reference sequence were obtained from the NCBI nonredundant protein database using Protein BLAST. After removing duplicated sequence from all homologous sequences,^31^ amino acid sequences with lengths between 250–800 were extracted. Sequences were clustered using CD-HIT,^32^ with a 90% homology threshold, and subjected to multiple alignment using MAFFT.^33^ After trimming regions of the sequence with an alignment gap of 10% or greater using trimAl,^34^ phylogenetic estimation was performed using the FastTree method.^35^

### Phylogenetic Analysis of Lactate Oxidase

Amino acid sequences were again obtained from the clade presumed to be that of LOX through a phylogenetic analysis of α-hydroxy acid oxidase. These sequences were reclustered using CD-HIT, with a homology threshold of 95%. After multiple alignment using MAFFT, regions in the sequence with alignment gaps of 10% or greater were trimmed using trimAl. Phylogenetic analysis was performed using IQ-TREE^36,37^ (1000 tree reconstruction replications plus bootstrapping. The amino acid substitution model used was LG+I+G4). Sequence homology in the phylogenetic tree was calculated using the Clustal Omega^38^ and heat map functions in R.^39^

### SDS-PAGE Analysis

The LOX solutions were treated with SDS-PAGE sample loading buffer (Thermo Fisher Scientific) and 100 mM dithiothreitol, then heated at 99 °C for 3 min. Next, the samples were subjected to Mini-PROTEAN TGX Stain-Free precast gel electrophoresis (Bio-Rad). Precision Plus Protein Unstained Protein Standards (Bio-Rad) were used as molecular weight markers, and bovine serum albumin (BSA, Takara, Japan) was used as the protein concentration standard. LOX concentrations were estimated by quantifying the band intensity using ImageJ^40^ and comparing it with the BSA band intensity.

### Enzyme Screening Assays

For the acidic pH tolerance LOX screening assay,^41^ 20 mM phosphate buffer (pH 7.0) and acidic artificial human sweat^42^ (0.5 g/L l-histidine hydrochloride monohydrate, 5.0 g/L sodium chloride, and 2.2 g/L sodium dihydrogen phosphate dihydrate) were used. Ten microliters of crude enzyme solution and 190 μL of reaction solution (1.5 mM 4-aminoantipyrine, 1.5 mM *N*-Ethyl-*N*-(2-hydroxy-3-sulfopropyl)-3-methylaniline (TOOS, Dojindo Laboratories, Japan), 2 U/mL horseradish peroxidase, and 5 mM l-lactate in each buffer) were mixed in a 96-well assay plate (AGC Techno Glass, Japan). Absorbance at 555 nm was measured at regular intervals at 37 °C (39.2 mM^–1^ cm^–1^ as the molar absorption coefficient of TOOS). One unit of oxidase activity was defined as the amount of enzyme producing 1 μmol of hydrogen peroxide per min at 37 °C. The relative activity (%) of each LOX (enzyme activity per 1 mg of protein) was calculated and compared to that of EfLOX.

### pH Profile Assay

The pH profiles of the enzymes were determined using the McIlvaine buffer (Fujifilm Wako, Japan). Ten microliters of enzyme solution, 100 μL of buffer solution, and 10 μL of 50 mM l-lactate were mixed in a 96-well assay plate and reacted at 25 °C. After 30 min, 40 μL of 1 mM 2,4-dinitrophenylhydrazine dissolved in 1 M hydrochloric acid solution was added to the mixture and incubated at room temperature (approximately 25 °C) for 10 min. Subsequently, 150 μL of 1 M sodium hydroxide was added to the mixture; after 5 min of incubation at room temperature, absorbance at 445 nm was measured. Pyruvate solution was used as the concentration standard. One unit of oxidase activity was defined as the pyruvate concentration (mM) converted from l-lactate by 1 mg of LOX in 1 min at 25 °C. The pH profile was defined as the relative enzyme activity (%) compared to the maximum oxidase activity at each pH.

### Fabrication of Screen-Printed Electrodes^17^

Printed electrodes were fabricated on Japanese paper (Shoun, Togawa Seishi, Japan) using a screen printer (LS-150TV, New-long Seimitsu Kogyo, Japan). Water-repellent (3.0 vol % concentration of NR-158, Nicca Chemical, Japan) and catalyst (0.6 vol % concentration of NY-80, Nicca Chemical) solutions were mixed at a 20:3 volume ratio. The Japanese paper was soaked in the water-repellent solution and allowed to dry at 25 °C for 24 h. The electro-conductive lead layers on the water-repellent Japanese paper were printed using carbon ink (JELCON CH-10, Jujo Chemical, Japan) and dried at 120 °C for 15 min; this process was repeated six times. Porous carbon ink was prepared by mixing 1 g of MgO-templated porous carbon (average pore diameter of 100 nm, CNovel MJ (3)­100, Toyo Tanso, Japan), 10.1 mL of polyvinylidene difluoride hexafluoropropylene copolymer (KF polymer L#9305, 5 wt % in 1-Methyl-2-pyrrolidone (NMP), Kureha, Japan), and 3.5 mL of NMP (Fujifilm Wako) in a planetary centrifugal mixer (Thinky, Japan) for 4 min. Thereafter, a porous carbon layer was printed on the lead layer and dried at 120 °C for 3 h; this process was repeated thrice. The apparent surface area of the porous carbon electrode was 1 cm^2^ (0.5 × 2.0 cm).

### Fabrication of Screen-Printed Electrodes Modified with LOX^17,43^

Before enzyme modification of the porous carbon electrode, as preliminary preparation, UV ozone treatment was performed for 60 min to remove impurities on the electrode surface. Thereafter, 20 μL of 50 mM 1,2-Naphthoquinone^43^ (Tokyo Chemical Industry, Japan) solution in acetonitrile was deposited on the porous carbon electrode and allowed to dry for 30 min at room temperature under vacuum conditions. The concentrations of LCO-301 (TOYOBO, Japan) and His-tagged LsLOX in 20 mM phosphate buffer (pH 7.0) were determined using SDS-PAGE. The LOX solution was dropped on the mediator-modified electrode so that approximately 127 μg of LOX was applied per electrode. The electrodes were dried for 90 min at room temperature under vacuum conditions.

### Electrochemical Measurement

Electrodes were evaluated via chronoamperometry (CA) using a potentiostat (VersaSTAT3, AMETEK) with a three-electrode system. An Ag/AgCl/saturated-KCl electrode and a Pt wire were used as the reference and counter electrodes, respectively. Measurements were conducted in McIlvaine buffer (pH 3.0, 4.0, 5.0, 6.0, and 6.8). A potential of +0.2 V was applied against the Ag/AgCl/saturated-KCl electrode. Aliquots of a 1 M sodium l-lactate solution were added during the measurement to achieve a stepwise increase in the concentration from 0 to 1, 3, 5, 10, and 15 mM (final concentrations). All measurements were performed thrice at 25 °C. The current density was calculated using the projected surface areas of the electrodes.

### Structural Analysis of LOX

The crystal structure data of AvLOX (PDB 2e77) and the predicted structure of LsLOX determined using Local ColabFold^44^ ver. 1.5.3 were visualized using the BIOVIA Discovery Studio software (Dassault Systems, France).

## Results

### Retrieval of Lactate Oxidase Sequences from Public Databases

In general, amino acid sequences registered in public databases have annotation information regarding their respective protein functions automatically predicted. However, differences exist in the annotation accuracies. Therefore, expressing proteins with annotated functions experimentally is not always possible. LOX has a limited number of sequences with experimentally confirmed functions. To search for new LOX from unannotated sequences, a search method that does not depend on annotation information is required.

First, we attempted to extract candidate LOX sequences from the phylogenetic trees of LOX, lactate dehydrogenase, and lactate monooxygenase. However, some sequences had ambiguous phylogenetic boundaries, which could have led to incorrect functional classification (data not shown). Therefore, we constructed a phylogenetic tree of α-hydroxy acid oxidases and established a method for obtaining new candidate sequences by referring to the phylogenetic position of sequences with reported lactate oxidation function.

Figure 1 shows a molecular phylogenetic tree of the α-hydroxy acid oxidase sequences registered in the NCBI database. Each clade was annotated based on the functions of the reference sequences. Four representative sequences^26,27,29,30^ were used as reference sequences for LOX. After searching for clades containing these four sequences, two clades were identified as shown in Figure 1d,e. Based on the results of this phylogenetic analysis, it was expected that the sequences contained in the two LOX clades would exhibit l-lactate oxidation activity.

**Figure 1:**
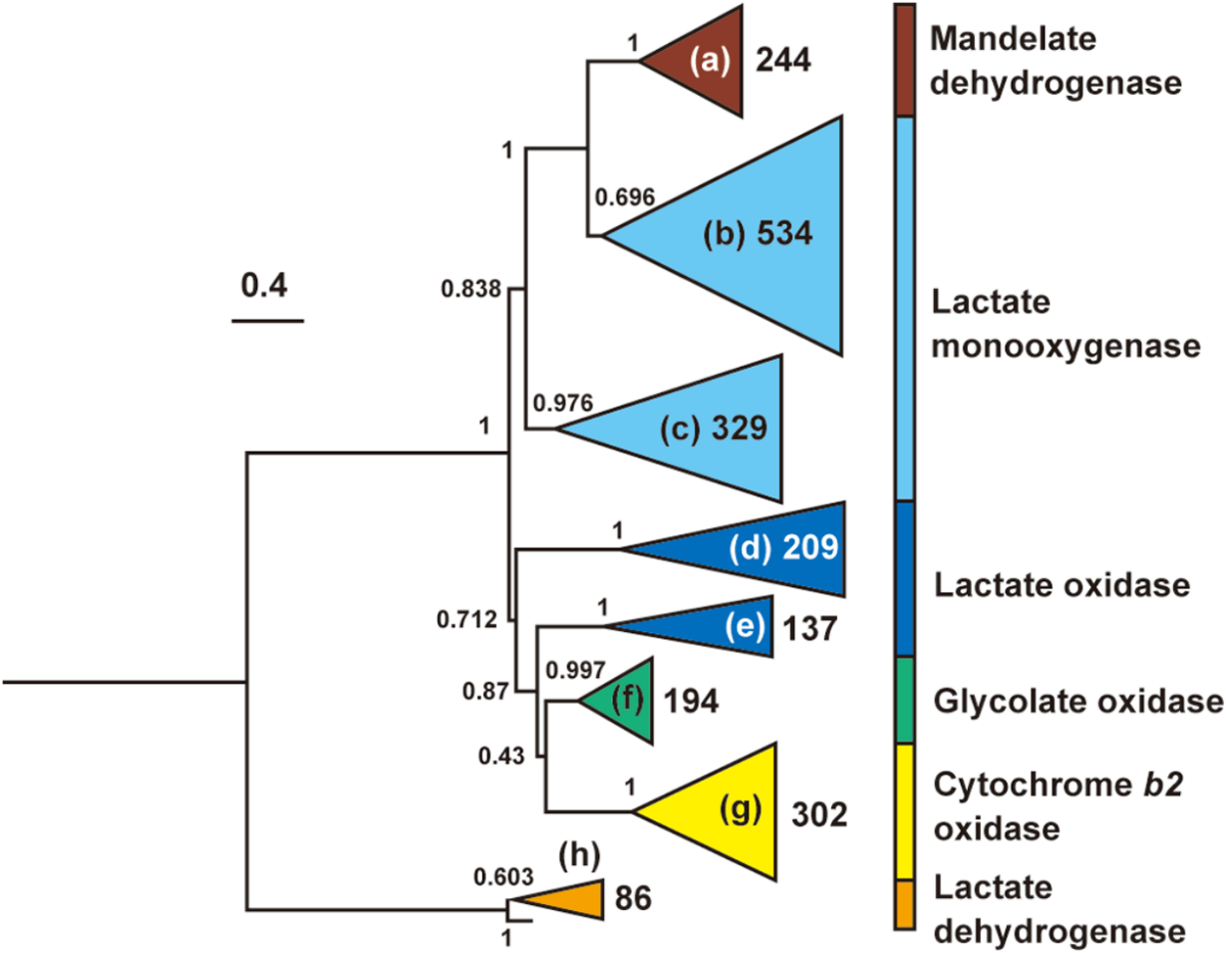
Phylogenetic tree of α-hydroxy acid oxidases with local support values calculated based on the Shimodaira–Hasegawa test. All sequences examined were classified into eight clades. Each clade was annotated according to the reference sequence. The number inside or next to the clade is the number of sequences therein.

### Selection of LOX Candidates

We obtained 346 LOX candidates from the phylogenetic tree of the α-hydroxy acid oxidases with 90% homology (Figure 1), including reference sequences. However, evaluating the acidic pH tolerance of all sequences was experimentally challenging. Therefore, we narrowed down the evaluation target by analyzing the phylogenetic relationships of the candidate sequences in more detail.

Figure 2 shows the results of phylogenetic analysis of candidate sequences with 95% homology. The tree was divided into three groups: sequences derived from bacteria (clade 1), eukaryotes (clade 2), and metagenomic DNA. We further classified both clades based on the reference sequences (1A–2D). Focusing on clade 1, we found that AvLOX^26^ and EfLOX,^27^ reported to have l-lactate oxidation activity, were located in clades 1A and 1B, respectively, and were phylogenetically close to each other. Contrastingly, LOX from Pediococcus acidilactici (PaLOX)^29^ was located in clade 1E and was phylogenetically distant from AvLOX and EfLOX. The crystal structure of PaLOX is almost homologous to that of AvLOX; however, its l-lactate oxidation activity has not been confirmed.^29^ Therefore, we adopted a strategy that focused on the selection of evaluation targets from clades 1A and 1B.

**Figure 2:**
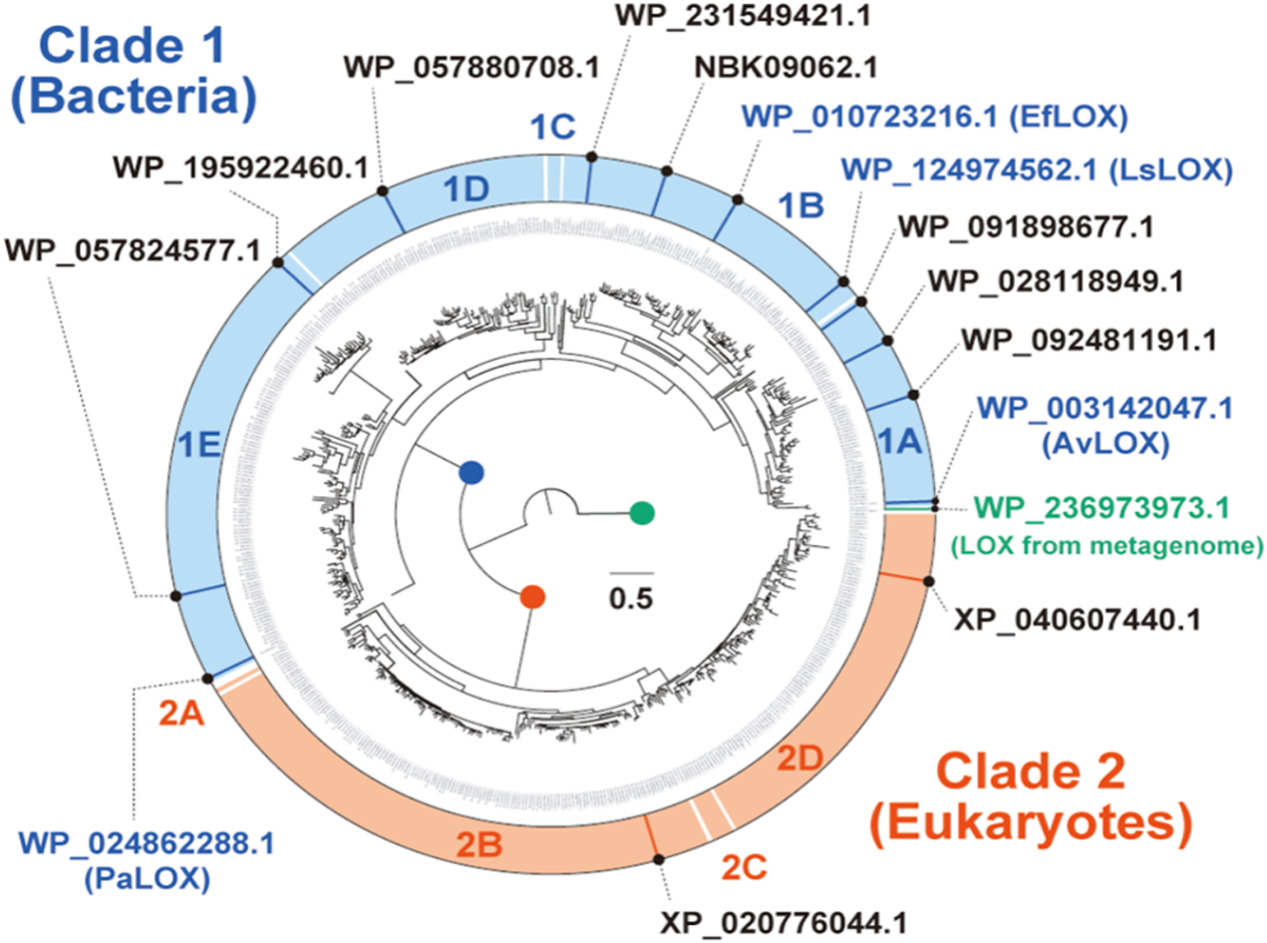
Phylogenetic analysis of l-lactic acid oxidase (LOX). The tree was divided into two clades, one derived mainly from bacteria (Clade 1) and another derived from eukaryotes (Clade 2).

Subsequently, we confirmed the sequence homology between each clade. Figure 3A shows the results of the multiple sequence alignment analysis carried out using Clustal omega, with the positional relationships of the phylogenetic tree preserved. Clade 1A is shown in Figure 3B and clade 1B is shown in Figure 3C. Three and five clusters in Figure 3B and Figure 3C were identified, respectively (indicated by the blue boxes in the figure). Among these relatively large clusters, three sequences with AvLOX homology between 61 and 75% in clade 1A, and three sequences with EfLOX homology between 71 and 88% in clade 1B, were selected as candidates. One sequence from each of the other clades was arbitrarily selected (AvLOX homology between 32 and 48% and EfLOX homology between 32 and 52%).

**Figure 3:**
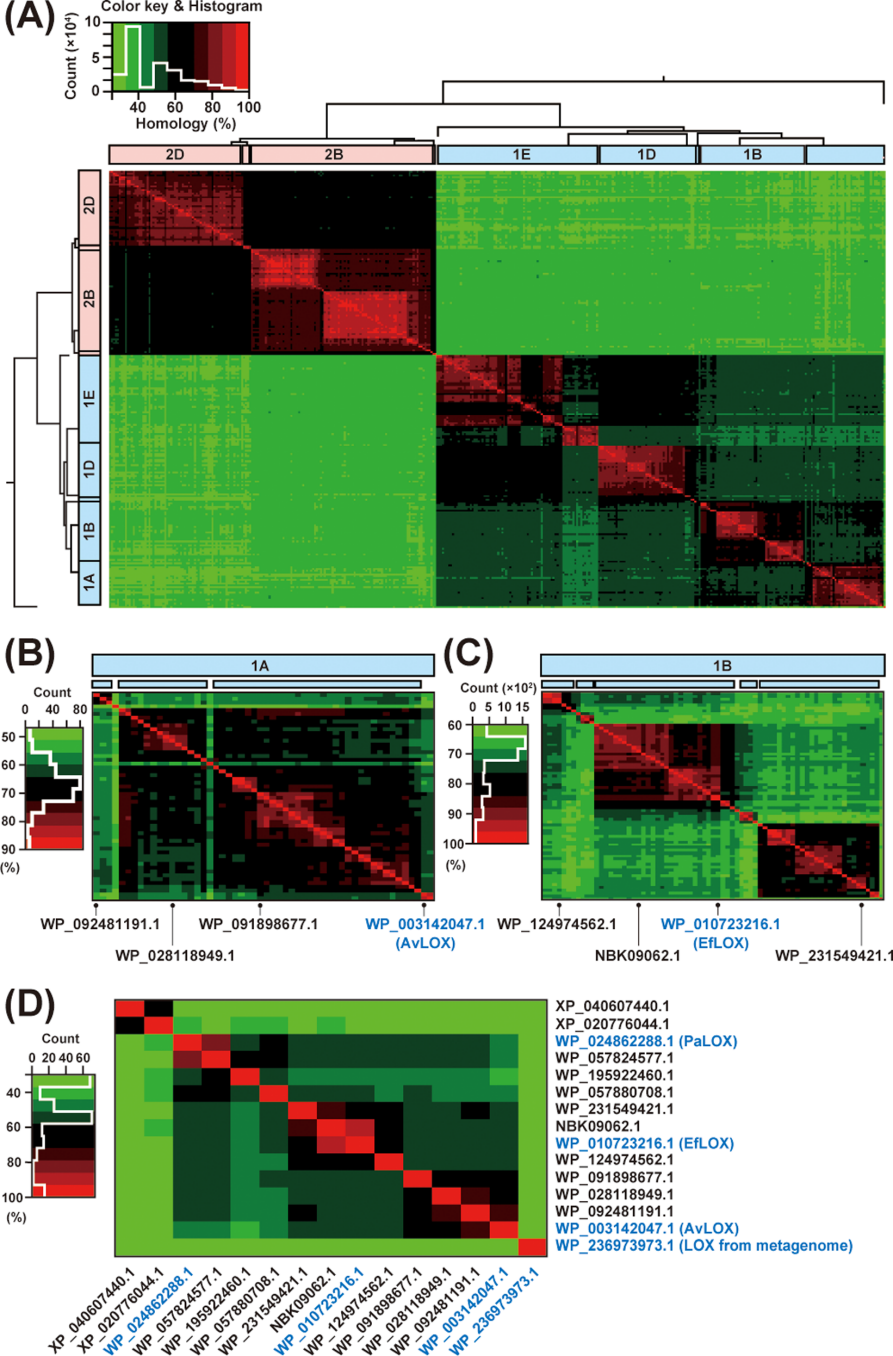
Visualization of LOX phylogenetic analysis results using Clustal Omega. (A) Homology heat map of the entire LOX phylogenetic tree (Figure 2). (B) Expanded homology heatmap of subclade 1A. (C) Expanded homology heatmap of subclade 1B. (D) Homology heat map of novel LOX candidate sequences.

### Screening for Novel Sequences with Acidic pH Tolerance

We evaluated the acidic pH tolerance of novel LOX candidates selected from the NCBI database. The candidate sequences were expressed in Escherichia coli (see Supporting Information) and soluble fractions of the cell lysates were prepared (Figure S1). l-lactate oxidation activity per protein of the crude enzyme was measured using 20 mM phosphate buffer (pH 7.0, Figure 4A) and acidic artificial human sweat solution (pH 5.5, Figure 4B). In this assay, both AvLOX and EfLOX were used as standards, with EfLOX showing increased activity in acidic artificial human sweat. Therefore, we used EfLOX as the benchmark enzyme in this study. Subsequently, by evaluating the activity of the new sequence in acidic artificial human sweat, we found that the sequence derived from LsLOX showed much higher activity than that derived from EfLOX (Figure 4B,C). However, the acidic pH was harsher than that observed under the optimal conditions associated with the modified Trinder’s reagent TOOS used for detection.^45^ Therefore, we prepared purified enzymes (see Supporting Information) with His-tag attached to the N-terminus of LsLOX and EfLOX (Figure S2) and evaluated their pH-activity profiles using 2,4-dinitrophenylhydrazine,^46^ as this method is less sensitive to acidic pH (Figure S3). We found that LsLOX exhibited greater acidic pH tolerance than that shown by EfLOX at pH 5.0, and surprisingly remained active even at pH 4.0 (Figure 4D), whereas EfLOX lost its activity at pH 4.0. Based on these findings, we concluded that LsLOX is a novel LOX with acidic pH tolerance.

**Figure 4:**
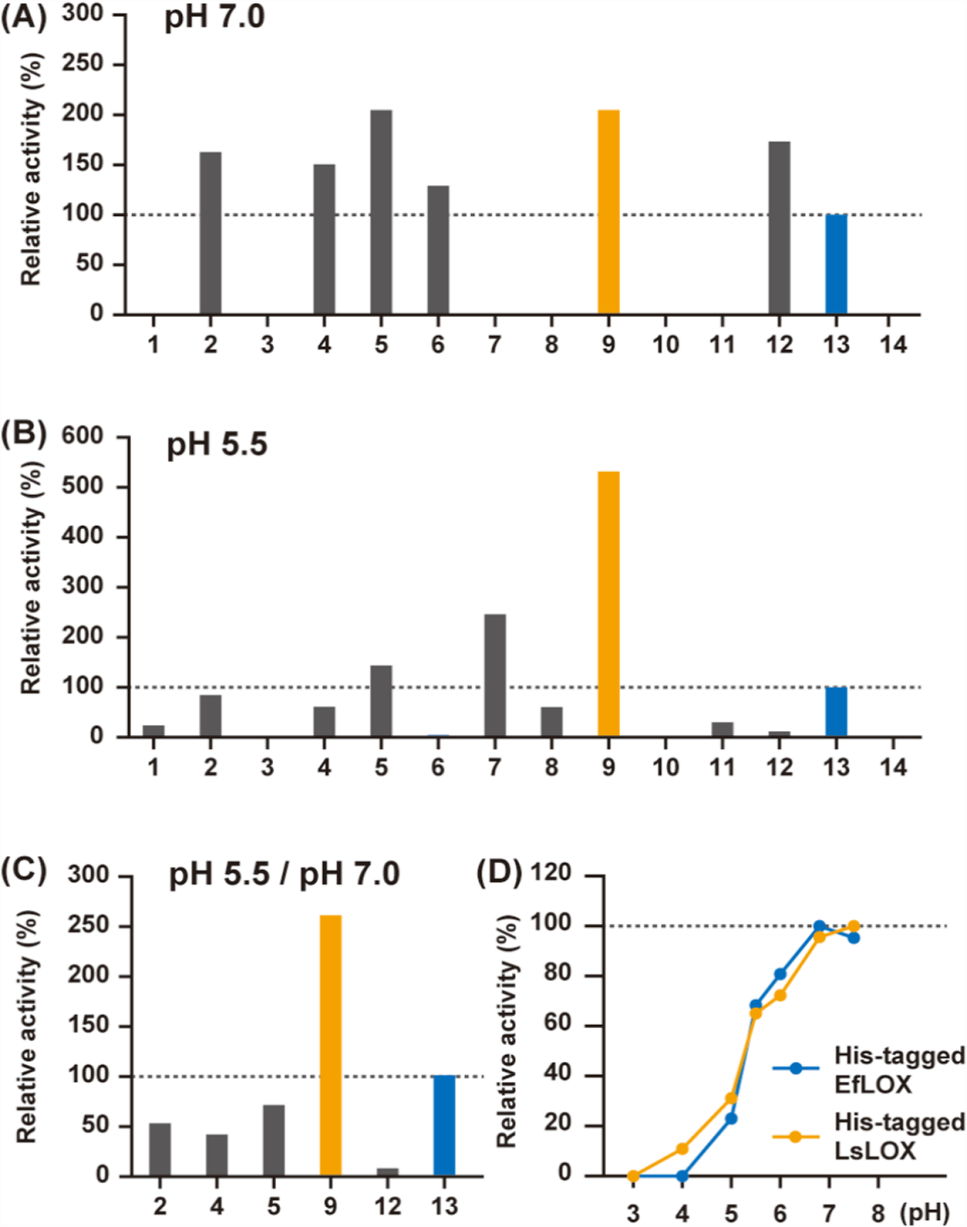
Screening of novel LOX with acidic pH tolerance. (A) Relative activity (%) of crude LOX in 20 mM phosphate buffer (pH 7.0). (B) Relative activity (%) of crude LOX in acidic artificial human sweat (pH 5.5). 1; XP_020776044.1, 2; WP_231549421.1, 3; WP_057880708.1, 4; WP_092481191.1, 5; NBK09062.1, 6; WP_028118949.1, 7; WP_195922460.1, 8; WP_057824577.1, 9; WP_124974562.1 (LsLOX), 10; WP_091898677.1, 11; XP_040607440.1, 12; WP_003142047.1 (AvLOX), 13; WP_010723216.1 (EfLOX), and 14; vector-negative control. (C) Comparison of the ratio of activity at pH 5.5 to pH 7.0. The relative value with EfLOX set to 100% is shown. LOX activity was measured using 4-aminoantipyrine, TOOS, and horseradish peroxidase. (D) Relative activity (%) of purified N-terminus His-tagged LOX in McIlvaine buffer. LOX activity was measured using 2,4-dinitrophenylhydrazine.

### Electrochemical Evaluation of LsLOX-Modified Electrode

Subsequently, a lactate biosensor was fabricated and evaluated electrochemically. For these proof-of-concept tests, a simple electrode design based on past studies was utilized.^17,43^ Cyclic voltammetry in the presence of l-lactate showed a catalytic response, especially at lower pH values (Figure S4). To test the sensitivity of the sensor to lactate concentrations, CA was conducted in McIlvaine buffer with l-lactate concentration adjusted stepwise from 0 to 15 mM (i.e., 0, 1, 3, 5, 10, and 15 mM), and the response of the sensor with increased lactate concentration was recorded (Figure S5). To verify the practical value of LsLOX, we used LCO-301, a commercially available LOX, for comparison. Figure 5 shows the relationship between the lactate concentration and increased current density. As shown in Figure 5C, electrode modified with 1,2-naphthoquinone/LCO-301 exhibited a linear relationship with lactate concentrations between 0 and 15 mM, as well as increased current density as the pH increased from 3.0 to 6.8. Conversely, as shown in Figure 5D, electrode modified with 1,2-naphthoquinone/LsLOX exhibited a linear relationship with pH, with almost the same slope observed at pH 3.0, 4.0, 5.0, and 6.0. In buffer at pH 6.8, LsLOX exhibited a linear relationship only from 0–1 mM. At higher concentrations, the response current increased slowly with increasing l-lactate concentration. Figure 5E shows a plot of the pH against sensor sensitivity in the linear range from 0 to 15 mM l-lactate; for LsLOX at pH 6.8, both the sensitivity from 0 to 1 mM l-lactate and that from 1 to 15 mM are shown. The sensitivity of LCO-301 shifted from 0.42 to 0.96 μA/mM as the pH increased from 3.0 to 6.8. In addition, the slope of the calibration line for LsLOX slightly decreased from 0.69 to 0.66 μA/mM as the pH increased from 3.0 to 6.0 (Figure S6). At pH 6.8, the sensitivity from 0 to 1 mM decreased slightly further to 0.62 μA/mM. At higher l-lactate concentrations, however, the sensitivity decreased to about a tenth (0.065 μA/mM, Figure S6). These data indicate that the sensitivity of LsLOX remained almost the same in the pH 3.0–6.0 range.

**Figure 5:**
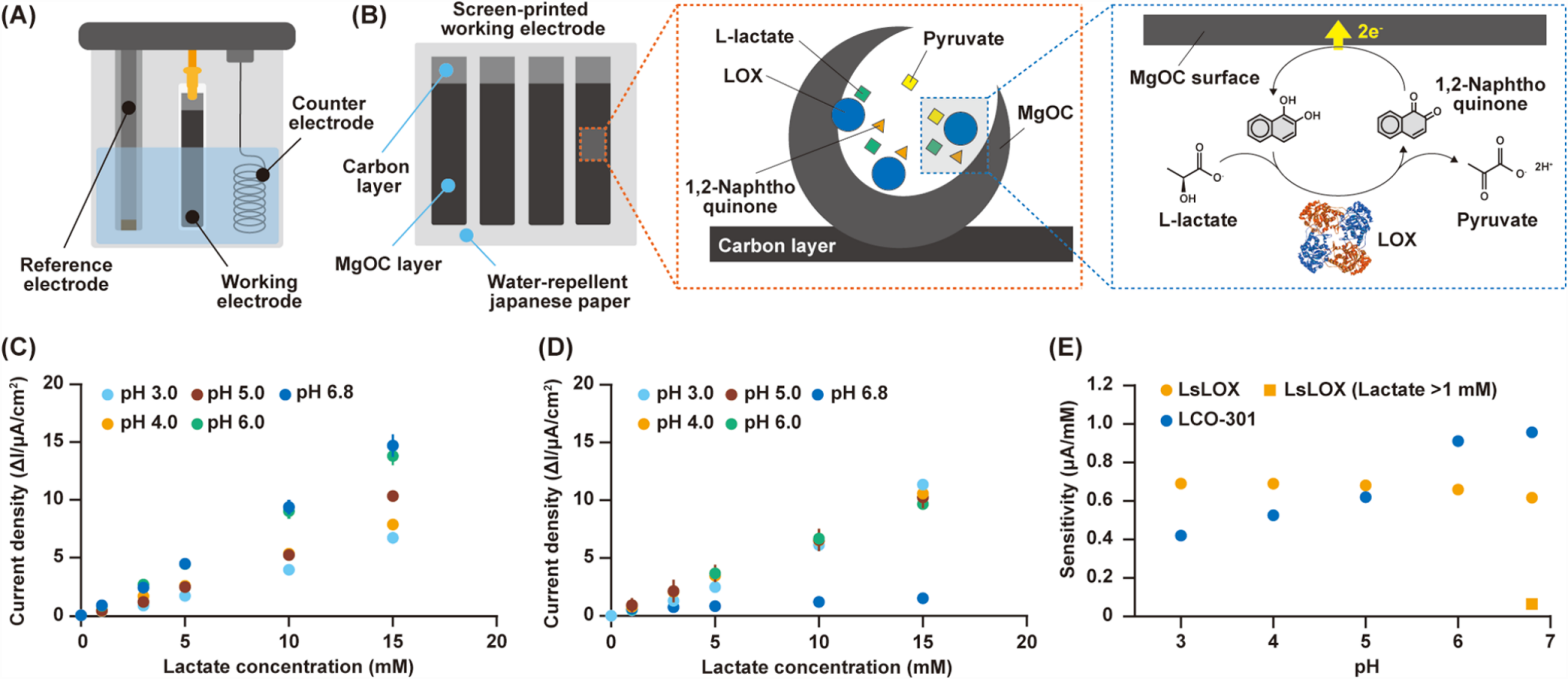
Electrochemical analysis of LOX from Enterococcus faecium (EfLOX) and LOX from Ligilactobacillus salitolerans (LsLOX). (A) Image illustrating the electrochemical evaluation. (B) Image depicting the working electrode component and the mechanism of current generation. The orange and blue dashed lines depict an enlarged view of the components and structure. (C, D) Correlation between lactate concentration and increased current density. Electrodes modified with (C) 1,2-naphthoquinone/LCO-301 and (D) 1,2-naphthoquinone/LsLOX were evaluated in McIlvaine buffer (pH 3.0, 4.0, 5.0, 6.0, and 6.8) containing 0-, 1-, 3-, 5-, 10-, and 15-mM l-lactate at 25 °C. For chronoamperometry, a potential of +0.2 V vs Ag/AgCl was applied (*n* = 3). (E) Correlation between pH and sensor sensitivity.

## Discussion

We identified a novel LOX showing improved acidic pH tolerance as compared to that shown by the conventional LOX. Surprisingly, we found electrodes with LsLOX exhibiting pH-independent l-lactate sensitivity. Analysis of the amino acid composition of the AvLOX, EfLOX, and LsLOX sequences showed minimal differences in the ratio of acidic and basic amino acids (Table S1). Subsequently, we performed multiple sequence alignments using the total lengths of the three sequences. The results suggested that important residues^47^ such as those involved in the localization of flavin mononucleotide (coenzyme), l-lactate, or pyruvate, were highly conserved; thus, no difference may have existed in the catalytic mechanism (Figure S7). However, the oligomeric structure only partially contributes to LOX activity. Furuichi et al.^48^ suggested the involvement of adjacent monomers in the hydrogen bonding network formed by the active residues of AvLOX. At neutral pH, AvLOX exists as a tetramer^47^ or an octamer;^49^ however, through a crystal structure analysis, Furubayashi et al.^50^ found that it exists as a dimer at pH 4.5. Collectively, these findings suggest that the stability of the oligomeric structure may partially contribute to its acidic pH tolerance. Ashok et al.^29^ showed that the length of the C-terminus is important for LOX oligomerization; therefore, we predicted the tetrameric structure of LsLOX using ColabFold.^44^ The predicted main chain structures of LsLOX and AvLOX were almost identical (Figure 6A). By contrast, LsLOX possesses a C-terminal sequence that is nine residues longer than that of AvLOX, and this predicted model showed that four residues in the C-terminal sequence interact with adjacent monomers (e.g., subunit A:Phe368-Subunit C:Phe43/Thr44, A:Asp373-C:Arg181, A:Lys375-C:Gln25, and A:Leu376-C:Lys30) (Figure 6B). We hypothesized that one possible reason for the acidic pH tolerance of LsLOX was the stability of its oligomeric structure, which involves its characteristically long C-terminus. A more detailed mechanistic analysis needs to be carried out in the future. LsLOX-modified electrodes showed almost identical responses to lactate in the pH range 3.0–6.0, while LsLOX in solution showed a clear pH dependency. The response currents of LCO-301-modified electrodes also decreased less than expected in acidic environments. This suggests that the tetrameric structure of LOX is further protected by the porous structure of MgOC. The double-protected tetrameric structure of the LsLOX in MgOC electrodes leads to the almost pH-independent sensitivity of the resulting biosensor. It should be noted, however, that the sensitivity of the LsLOX electrode at pH 6.8 to l-lactate concentrations higher than 1 mM was significantly decreased. This is in contrast to the enzyme’s activity in solution, which increased with increasing pH. While further investigations are needed to determine the cause for this unusual behavior, it seems reasonable to assume that at pH 6.8 and higher l-lactate concentrations, LsLOX transfers electrons preferentially to molecular oxygen and not to 1,2-naphthoquinone. The redox potential of quinones is known to shift to more negative values with increasing pH.^51,52^ A more positive redox potential of LsLOX compared to other LOXs, therefore, could explain an impeded electron transfer to 1,2-naphthoquinone at pH 6.8 not observed with other LOXs. A more suitable mediator, with a more positive redox potential at neutral pH values, might result in a LsLOX electrode with higher sensitivity at pH 6.8. Additionally, the change in sensitivity at 1 mM l-lactate could be explained by a change in structure due to bound l-lactate or pyruvate. Again, further investigations are needed to confirm these hypotheses. Nevertheless, one practical consequence, apparent from the results in this study, is that a LsLOX-based biosensor can be applied to determine lactate concentrations in samples with various and especially varying pH, such as for the continuous measurement of lactate in human sweat during exercise. Only one calibration at one pH is needed for a fully developed LsLOX-based lactate biosensor, making the sensor quasi-calibration-free. While further investigations are needed to verify sensor stability and selectivity, the structural similarity of the main chains of LsLOX and AvLOX suggests that similar results can be expected.

**Figure 6:**
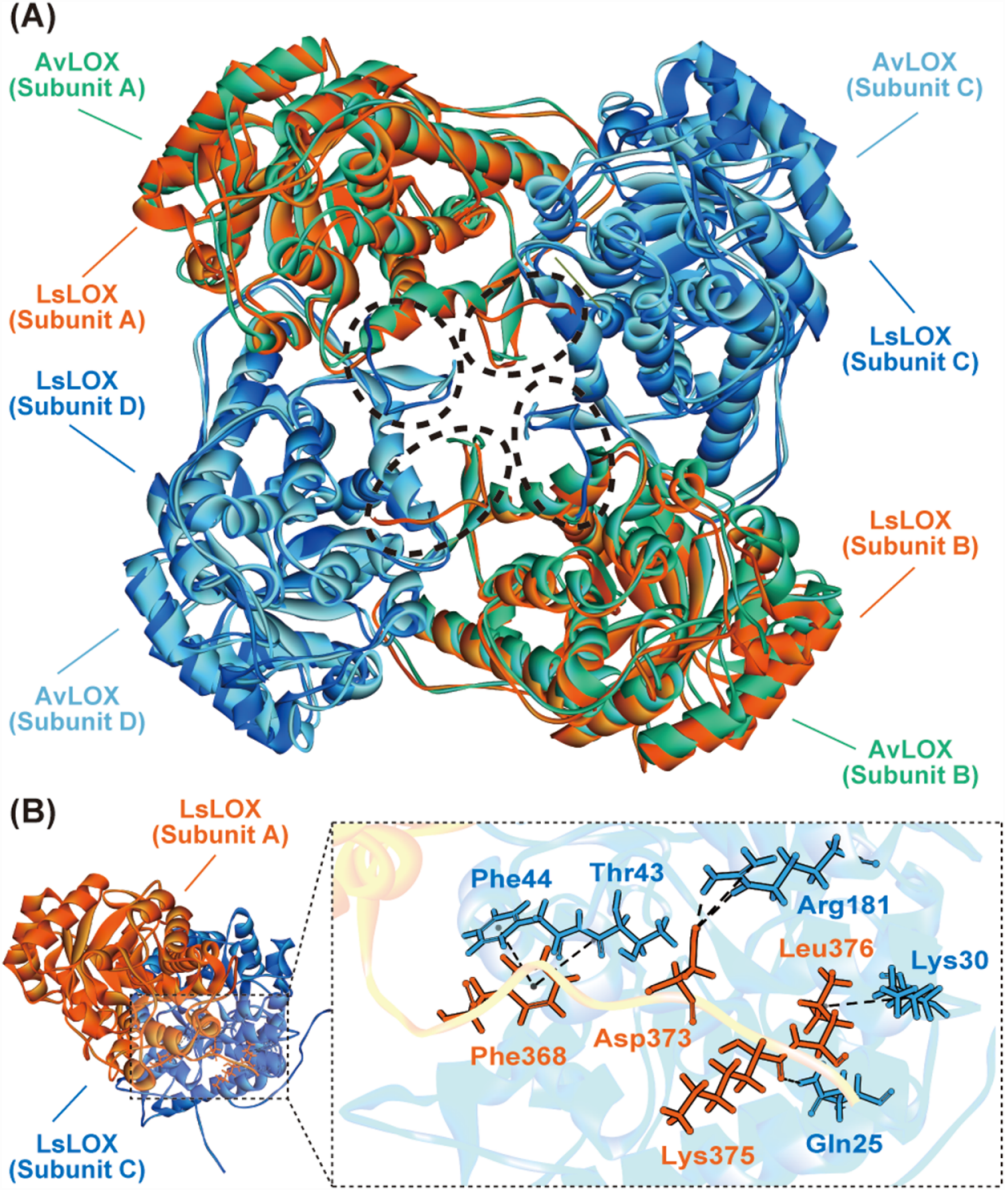
Comparison of 3D structure between LOX from Aerococcus viridans (AvLOX) and LOX from Ligilactobacillus salitolerans (LsLOX). (A) Superimposed structure of AvLOX and LsLOX. The name of the LsLOX subunit is represented as AvLOX (PDB 2e77) for convenience. The C-terminal sequences are highlighted using black dotted circles. (B) Enlarged view of the interaction between the C-terminus monomer (subunit A) and the adjacent monomer (subunit C) of the LsLOX tetramer structure. Predicted interactions are depicted using black dotted lines.

## Conclusions

In this study, we successfully identified a novel LOX with acidic pH tolerance using a public protein database. LsLOX exhibited greater acidic pH tolerance than exhibited by the conventional AvLOX and EfLOX. Surprisingly, at pH 4.0, which is commonly encountered in human acidic sweat, LsLOX showed more than 10% residual activity, whereas EfLOX showed no activity. Commercially available LOX (LCO-301)- and LsLOX-modified electrodes were prepared by screen-printing MgO-templated carbon on Japanese paper. The LCO-301- and LsLOX-modified electrodes exhibited an increased linear current density as the l-lactate concentration increased from 0 to 15 mM. The sensitivity of the LCO-301-modified electrode decreased as the pH changed from neutral to acidic, whereas the LsLOX-modified electrode exhibited constant sensitivity independent of pH. This finding suggests that the LsLOX-modified electrode could provide data-calibration-free technology for l-lactate sensing in samples with varying pH. In other words, a sensor utilizing LsLOX can be used to accurately quantify l-lactate in samples with different or varying pH with a single calibration. As an individual’s baseline sweat pH can be quite acidic depending on their regular dietary and/or exercising routines, this study contributes to the practical application of continuous, noninvasive l-lactate biosensing in sports, medicine, and nursing care fields as well as in food manufacturing and biotechnology industries.

## Supplementary Material



## Notes

Construction of LOX expression plasmid DNA and preparation of LOX; construction of His-tagged LOX expression plasmid DNA and preparation of His-tagged LOX; SDS-PAGE of LOX expression in E. coli (Figure S1); SDS-PAGE of His-tagged LOX (Figure S2); measurement system using 2,4-nitrophenyl hydradine capable of constant pyruvate detection at acidic to neutral pH (Figure S3); cyclic voltammograms of the electrode modified with 1,2-naphthoquinone/LsLOX (Figure S4); chronoamperometry curves of the electrode modified with 1,2-naphthoquinone/LsLOX (Figure S5); correlation between lactate concentration and increased current density (Figure S6); multiple sequence alignment of AvLOX, EfLOX and LsLOX (Figure S7); comparison of acidic and basic amino acid composition of AvLOX, EfLOX and LsLOX (Table S1); amino acid sequences of LOX (Sequence S1) (PDF)

## Abbreviations

LOX: l-lactic acid oxidase
AvLOX: LOX from Aerococcus viridans
EfLOX: LOX from Enterococcus faecium
LsLOX: LOX from Ligilactobacillus salitolerans
BSA: bovine serum albumin
NMP: 1-Methyl-2-pyrrolidone
CA: chronoamperometry
PaLOX: LOX from Pediococcus acidilactici
