# Supplementary material for: l‑Lactate Oxidase-Based Biosensor Enables Quasi-Calibration-Free Detection of l‑Lactate in Sweat of Acidic to Neutral pH
Source: ACS Sens. 2025 Jun 6;10(6):4707–16. doi: 10.1021/acssensors.5c01238 (PMC12210267; doi:10.1021/acssensors.5c01238)
Supplement: Supplementary file 1 [file se5c01238_si_001.pdf]

Supporting Information

L-Lactate Oxidase-Based Biosensor Enables Quasi-Calibration-Free Detection of L-Lactate in Sweat of Acidic to Neutral pH

Kosuke Ike[1*], Kousuke Muto[2], Takahiro Hioki[1], Noya Loew[3], Isao Shitanda[3*], Masafumi Takesue[2] and Mitsuyoshi Okuda[4]

[1]Laboratory of Biological & Material Science Research, Kao Corporation, 1334 Minato, Wakayama, 640-8580, Wakayama, Japan

[2]Laboratory of Performance Chemicals Research, Kao Corporation, 1334 Minato, Wakayama, 640-8580, Wakayama, Japan

[3]Department of Pure and Applied Chemistry, Faculty of Science and Technology, Tokyo University of Science, 2641 Yamazaki, Noda 278-8510, Chiba, Japan

[4]Laboratory of Biological & Material Science Research, Kao Corporation, 2606 Akabane, Ichikai, Haga, 321-3497, Tochigi, Japan

* E-mail: ike.kousuke@kao.com, shitanda@rs.tus.ac.jp

Material and Method

Construction of LOX expression plasmid DNA and preparation of LOX

Construction of His-tagged LOX Expression Plasmid DNA and Preparation of His-tagged LOX

Figure S1. SDS-PAGE of LOX expression in *E. coli*

Figure S2. SDS-PAGE of His-tagged LOX

Figure S3. Measurement system using 2,4-nitrophenyl hydradine capable of constant pyruvate detection at acidic to neutral pH

Figure S4. Cyclic voltammograms of the electrode modified with 1,2-naphthoquinone/LsLOX

Figure S5. Chronoamperometry curves of the electrode modified with 1,2-naphthoquinone/LsLOX

Figure S6. Correlation between lactate concentration and increased current density

Figure S7. Multiple Sequence Alignment of AvLOX, EfLOX and LsLOX

Table S1. Comparison of Acidic and Basic Amino Acid Composition of AvLOX, EfLOX and LsLOX

Sequence S1. Amino Acid Sequences of LOX

Material and Method

Construction of LOX expression plasmid DNA and preparation of LOX

Coding sequences (CDS) of L-lactate oxidase (Sequence S1) were optimized for *E. coli* codon usage by GenScript Japan. pET22b(+) was used as the backbone of the plasmid for protein expression. The expression vector was synthesized in GenScript Japan as an artificial gene so that the gene sequence would replace the sequence from the start of the *pel*B signal sequence to just before the T7 terminator. The plasmid DNA for LOX expression was transformed into ECOS™ Competent E.coli BL21(DE3) (Nippon Gene, Japan) by heat-shock. Positive clones were selected on LB medium (SERVA, Germany) with 1% agar and 100 μg/mL ampicillin. Colonies were cultured in 2 mL LB liquid medium (SERVA) with 100 μg/mL ampicillin at 30 °C overnight. Twenty microliters of the seed were inoculated in 2 mL of Overnight Express™ Instant TB Medium (Sigma-Aldrich, USA) with 100 μg/mL ampicillin and then cultured at 30⊠, 20 hours. The cultured cells were washed twice using saline (150 mM sodium chloride, 10 mM MOPS). The cells were resuspended in 2 mL of lysis buffer (20 mM phosphate buffer (pH 7.0), 10% BugBuster® Master Mix (Merck Millipore, USA), 1% Protease Inhibitor Cocktail Set VII (Fujifilm Wako, Japan)). The cells were lysed using Multi-Beads Shocker (bead diameter 0.5 mm, Yasui instrument, Japan) under 4 °C condition. The soluble fraction after centrifugation was used as a crude enzyme solution.

Construction of His-tagged LOX Expression Plasmid DNA and Preparation of His-tagged LOX

Amplification of plasmid DNA with 6×His-tag at the N-terminus of the CDS was performed by inverse PCR using the plasmid DNA mentioned above as template and the following primers: the forward primers (EfLOX: 5'-CATCACCATCACCACATGGAGAAAACGTACCAGGC-3', LsLOX: 5'-CATCACCATCACCACATGGGGCATTTAAAACATCAAGTG-3') and the reverse primer (5'-ATGGTGATGATGCATATGTATATCTCCTTCTTAAAGTTAAAC-3'). The PCR reaction was performed under the recommended conditions of KOD One® PCR Master Mix-Blue- (TOYOBO, Japan). PCR products were purified using NucleoSpin® Gel and PCR Clean-up (Macherey-Nagel, Germany) and cloned using In-fusion HD Cloning Kit (Takara, Japan). This DNA was transformed into ECOS™ Competent E.coli DH5α (Nippon Gene) by heat-shock, and positive clones were selected on LB medium with 1% agar and 100 μg/mL (final) ampicillin. Plasmid DNA was extracted from *E. coli* cultured in 100 μg / mL (final) ampicillin LB liquid medium using NucleoSpin® Plasmid EasyPure (Macherey-Nagel).

Plasmid DNA for His-tagged LOX expression was transformed into ECOS™ Competent E.coli BL21(DE3) (Nippon Gene) for EfLOX or DynaCompetent® Cells BL21(DE3) pLysS (BioDynamics Laboratory, USA) for LsLOX by heat-shock .

One milliliter of *E. coli* seed solution expressing His-tagged LOX was transplanted into 100 mL of Overnight Express™ Instant TB Medium with 100 μg/mL (final) ampicillin in a baffled flask and the main culture (10 flasks per an enzyme) was carried out at 37 °C for 24 h. The cells were washed twice using saline and then resuspended in 100 mL of 20 mM phosphate buffer (pH7) at 4 °C and ultrasonically lysed using Bioruptor II (BM Equipment, Japan). The lytic solution was centrifuged, and the soluble fraction was filtered using a 0.2 μm-pore size membrane (ThermoFisher Scientific, USA). The filtrate was adsorbed with Ni-NTA agarose (Fujifilm Wako) for 2 h. Ni-NTA agarose was washed twice using wash buffer (0.1 M phosphate buffer (pH 8.0), 0.5 M sodium chloride, 10 mM imidazole) and then eluted with 100 mL of elution buffer (0.75 M

phosphate buffer (pH 6.5), 0.5 M sodium chloride, 0.5 mM imidazole). Eluates containing purified enzymes were buffer-exchanged and concentrated using Amicon Ultra-15 (Merck, Germany) to obtain LOX purified enzymes dissolved in 1 mL of 20 mM phosphate buffer (pH 7.0).

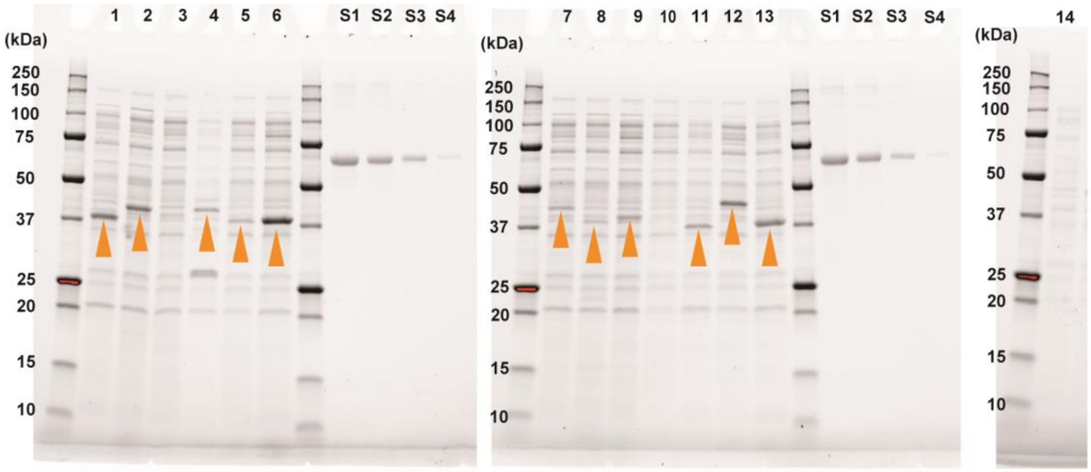

Figure S1. SDS-PAGE of LOX expression in *E. coli.* (1) XP_020776044.1, (2) WP_231549421.1, (3) WP_057880708.1, (4) WP_092481191.1, (5) NBK09062.1, (6) WP_028118949.1, (7) WP_195922460.1, (8) WP_057824577.1, (9) WP_124974562.1 (LsLOX), (10) WP_091898677.1, (11) XP_040607440.1, (12) WP_003142047.1 (AvLOX), (13) WP_010723216.1 (EfLOX) and (14) vector negative-control. BSA standards are shown in (S1) - (S4). (S1) 1500 ng, (S2) 1000 ng, (S3) 500 ng and (S4) 100 ng per lane.

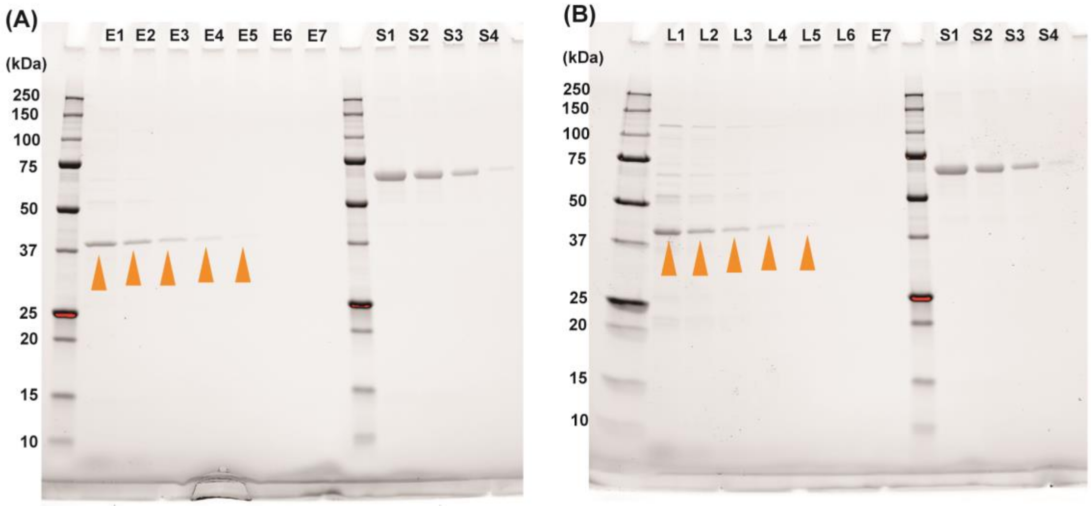

Figure S2. SDS-PAGE of His-tagged LOX. (A) EfLOX. (E1) is a 1/50 dilution sample, and (E2) and subsequent samples are further diluted by 1/2. (B) LsLOX. (L1) is a 1/50 dilution sample, and (L2) and subsequent samples are further diluted by 1/2. BSA standards are shown in (S1) - (S4). (S1) 1500 ng, (S2) 1000 ng, (S3) 500 ng and (S4) 100 ng per lane.

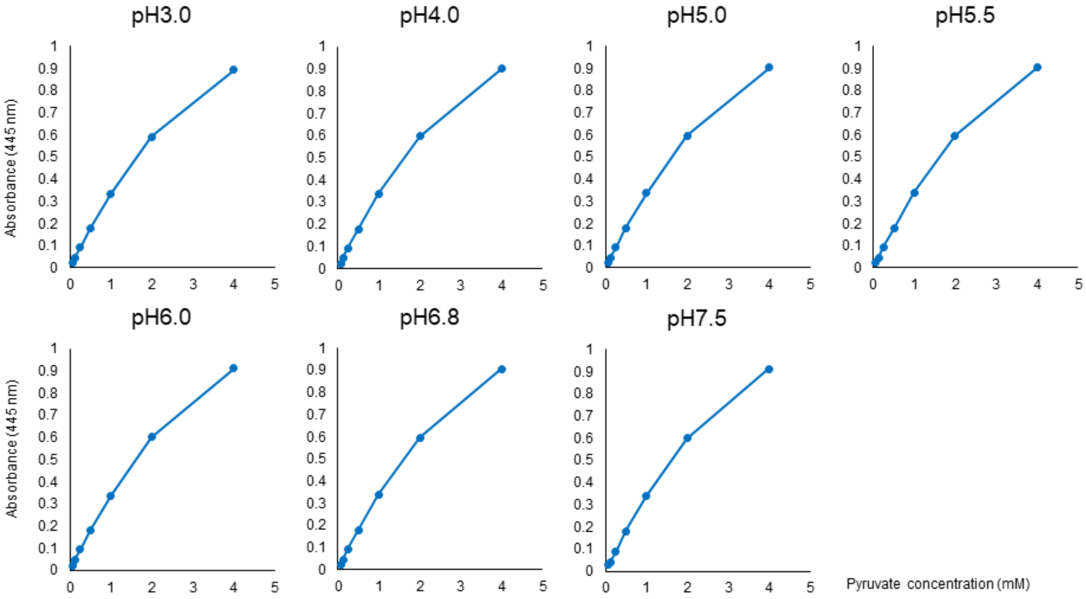

Figure S3. Measurement system using 2,4-nitrophenyl hydradine capable of constant pyruvate detection at acidic to neutral pH.

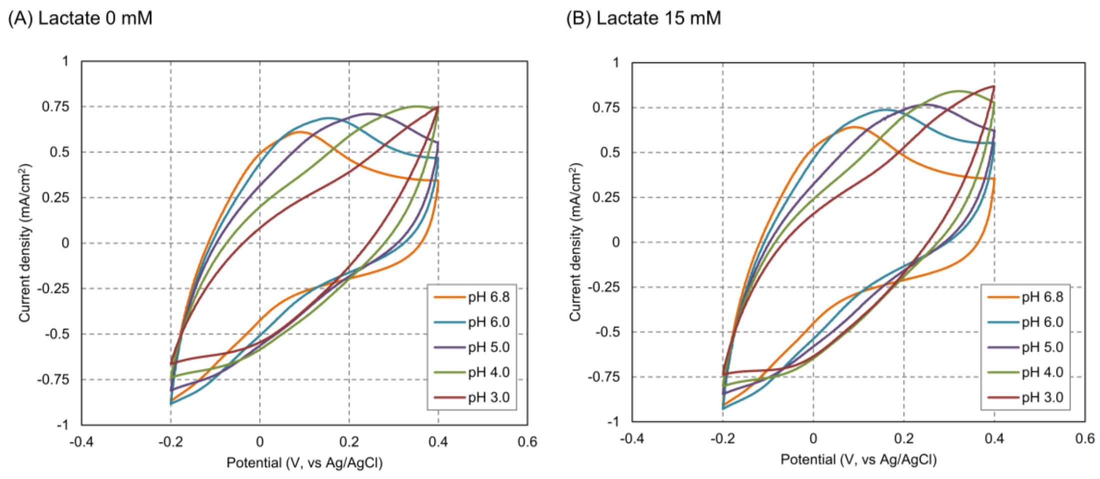

Figure S4. Cyclic voltammograms of the electrode modified with 1,2-naphthoquinone/LsLOX. (A) Lactate concentration is 0 mM, and (B) lactate concentration is 15 mM in McIlvaine buffer.

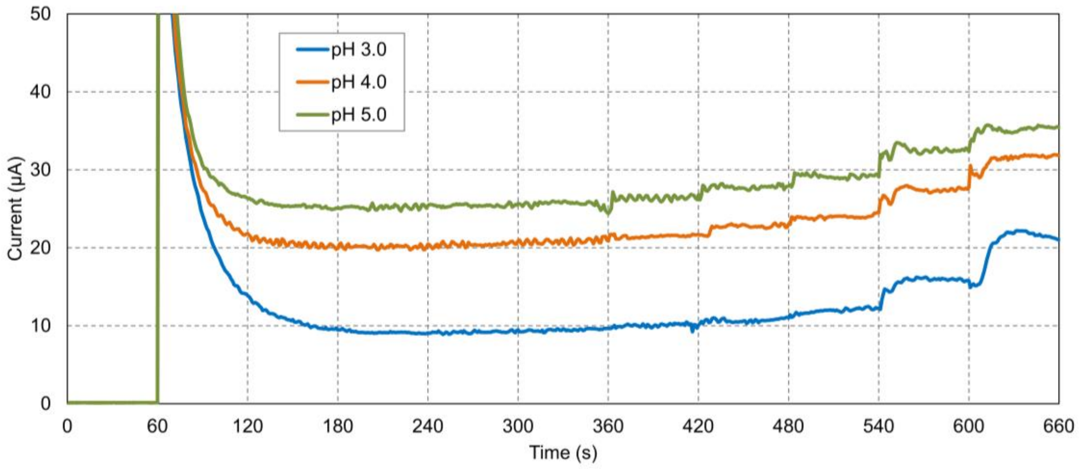

Figure S5. Chronoamperometry curves of the electrode modified with 1,2-naphthoquinone/LsLOX. Current was measured in McIlvaine buffer and voltage of 0.2 V. Lactate was added every 60 seconds starting at 360 seconds.

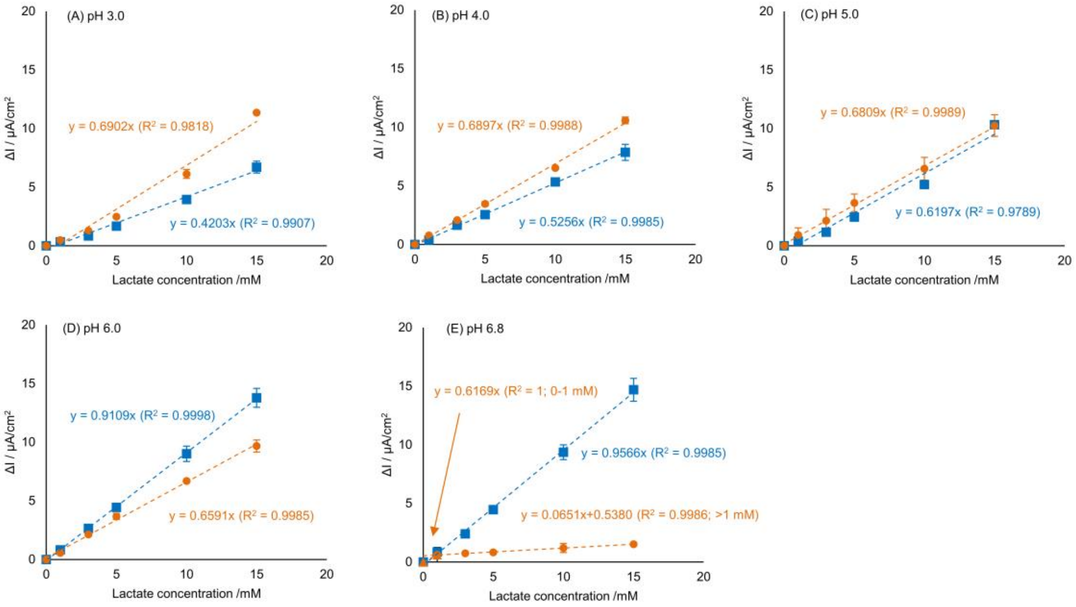

Figure S6. Correlation between lactate concentration and increased current density. Electrodes modified with 1,2-naphthoquinone/LCO-301 (blue) and 1,2-naphthoquinone/LsLOX (orange) were evaluated in McIlvaine buffer pH (A) 3.0, (B) 4.0, (C) 5.0, (D) 6.0, and (E) 6.8.

```
                10         20         30         40         50         60         70         80
AvLOX MNNNDIEYNA PSEIKYIDVV NTYDLEEEAS KVVPHGGFNY IAGASGDEWT KRANDRAWKH KLLYPRLAQD VEAPDTSTEI
EfLOX ---MEKTYQA GTNEGIVDFI NMEDLEIAAS QVIPAGGYGY ISSGAGDLFT YQENERAFNH QLIIPHVLRD VELPDTTTHF
LsLOX -----MAFKT SSAEGPVDFI NTFDLEKMAQ KVIPKGAFGY IASGAGDTFT LHENIRSFNH KLIVPHTLMD VEEPETKTIF

                90        100        110        120        130        140        150        160
AvLOX LGHKIKAPFI MAPIAAHGLA HTTKEAGTAR AVSEFGTIMS ISAYSGATFE EI-SEGLNGG PRWFQIYMAK DDQQNRDILD
EfLOX DKETLTAPII MAPVAAHGLA HVKAEKASAK GVADFGTIYT ASSYASCTLE EIREAGGEKA PQWFQFYMSK DNGINLDILE
LsLOX AGEKLTAPMI MAPVAAHKLA NEQGETASAK GVADFGSIYT LSSYSSVDLP EV-TAALHGA PEWFQFYFSK DNGINRHIMD

               170        180        190        200        210        220        230        240
AvLOX EAKSDGATAI ILTADSTVSG NRDRDVKNKF VYPFGMPIVQ RYLRGTAEGM SLNNIYGASK QKISPRDIEE IAAHSGLPVF
EfLOX VAKRNGAKAI VLTADATVGG NRETDRRNGF TFPLPMPIVQ AY--QSGVGQ TMDAVYKSSK QKLSPKDVEF IAAHSDLPVY
LsLOX RVKEQGIKAI VLTADAMVGG NRETDARNNF TFPVTLPIVQ EYL-PDSVGK TMDYVYKSAK QKLSPKDVEF IAEYSGLPVY

               250        260        270        280        290        300        310        320
AvLOX VKGIQHPEDA DMAIKAGASG IWVSNHGARQ LYEAPGSFDT LPAIAERVNK RVPIVFDSGV RRGEHVAKAL ASGADVVALG
EfLOX VKGVQSEEDV YRSLESGAGG IWVSNHGGRQ LDGGPAAFDS LQYVAEAVDK RVPIVFDSGV RRGQHVFKAI ASGADLVAIG
LsLOX VKGPQSKEDV DRALSAGAAG IWVSNHGGRQ LDGGPAAFDS LQEVAQAVDH RVPVIFDSGI RRGQHIFKAI ASGADLVALG

               330        340        350        360        370        380
AvLOX RPVLFGLALG GWQGAYSVLD YFQKDLTRVM QLTGSQNVED LKGLDLFDNP YGYEY----- ----
EfLOX RPVIYGLSLG GSTGVHQVFD FFKTELEMVM QLAGTQTVED IKKIKLRENR FI-------- ----
LsLOX RPVIYGLGLG GSVGVKQVFE KLQAELKLVM QLAGTQTVED IKNFNLRDNH YDPAFDTSHD LKLV
```

Figure S7. Multiple Sequence Alignment of AvLOX, EfLOX and LsLOX. Residues conserved in three sequences are shown in red, and residues conserved in two sequences are shown in green. Residues highlighted in red indicate those considered important for L-lactate oxidative activity in AvLOX.[30]

Table S1. Comparison of Acidic and Basic Amino Acid Composition of AvLOX, EfLOX and LsLOX. The numbers represent the number of amino acids and their ratio (%) to the full sequence length.

	AvLOX	EfLOX	LsLOX
Asp	26 (6.95%)	22 (5.99%)	25 (6.63%)
Glu	23 (6.15%)	24 (6.54%)	21 (5.57%)
Arg	18 (4.81%)	15 (4.09%)	12 (3.18%)
His	9 (2.41%)	9 (2.45%)	11 (2.92%)
Lys	20 (5.35%)	20 (5.45%)	25 (6.63%)

Sequence S1. Amino Acid Sequences of LOX

>WP_003142047.1 L-lactate oxidase [Aerococcus viridans]

MNNNDIEYNA PSEIKYIDVV NTYDLEEEAS KVVPHGGFNY IAGASGDEWT KRANDRAWKH KLLYPRLAQD

VEAPDTSTEI LGHKIKAPFI MAPIAAHGLA HTTKEAGTAR AVSEFGTIMS ISAYSGATFE EISEGLNGGP

RWFQIYMAKD DQQNRDILDE AKSDGATAII LTADSTVSGN RDRDVKNKFV YPFGMPIVQR YLRGTAEGMS

LNNIYGASKQ KISPRDIEEI AAHSGLPVFV KGIQHPEDAD MAIKAGASGI WVSNHGARQL YEAPGSFDTL

PAIAERVNKR VPIVFDSGVR RGEHVAKALA SGADVVALGR PVLFGLALGG WQGAYSVLDY FQKDLTRVMQ

LTGSQNVEDL KGLDLFDNPY GYEY

>WP_010723216.1 alpha-hydroxy-acid oxidizing protein [Enterococcus faecium]

MEKTYQAGTN EGIVDFINME DLEIAASQVI PAGGYGYISS GAGDLFTYQE NERAFNHQLI IPHVLRDVEL

PDTTTHFDKE TLTAPIIMAP VAAHGLAHVK AEKASAKGVA DFGTIYTASS YASCTLEEIR EAGGEKAPQW

FQFYMSKDNG INLDILEVAK RNGAKAIVLT ADATVGGNRE TDRRNGFTFP LPMPIVQAYQ SGVGQTMDAV

YKSSKQKLSP KDVEFIAAHS DLPVYVKGVQ SEEDVYRSLE SGAGGIWVSN HGGRQLDGGP AAFDSLQYVA

EAVDKRVPIV FDSGVRRGQH VFKAIASGAD LVAIGRPVIY GLSLGGSTGV HQVFDFFKTE LEMVMQLAGT

QTVEDIKKIK LRENRFI

>WP_092481191.1 lactate oxidase [Desemzia incerta]

MAEQTIKYDA PTVEQEIEVV STYRLEDQAR AVVPKGGFDY ISGASGEEYT LKQNNESWKN KGILPRVLAD

VENPDTSTSI LGHDIKVPFI MAPIAAHGLA HETKEAGTAR GISEFGGTIM SISAYSGATF EEIEKGLNGN

PRWFQIYMSK DDDMNKNILD EAKADGATAI ILTADATLSG NREKDLLNKF VYPFGMPIVS RYLTGSGKNM

SLNNIYAQSK QKICPADVKF IAEYSGLPVF VKGIQTPEDA VLAIGAGASG VWVSNHGGRQ LDEAPGSFDT

LEEIAKAVAG RVPIVFDSGV RRGEHIFKAL ASGADIVAVG RPVLYGLALG GWKGVKSVLD YFEKDLKRVM

QLAGTQTIED VKNARLFDMK K

>WP_028118949.1 lactate oxidase [Hutsoniella sourekii]

MTQDKYLAPS EVKPLRIINT IELEDMASEI VPHGGFNYMA GGSGDEFTLR RNVESFNFKG ILPRMAADVE

FPETDTKIFD HHLKVPFIMA PIAAHGLAHE SKEAGTARGI HEFGGSLMSI SAYSGASFEE ISAGLQDTPR

WFQIYMSKDD DFNRNILDEA KADGASAIIL TADATISGNR DRDDKNEFVY PFGMPIVSRY LTGTGANMSL

NNIYSQSKQK INLDDIRFIK EYSGLPVFLK GVQSPEDALA AIGAGADGIW VSNHGGRQLD GAPGSFEVLE

EIAQAVAGQV PIVFDSGIRR GEHIFKALAS GADIVALGRP VLFSLALGGW QGVQSVFEYF ERDLKRVMQL

AGTQTIEDVK KARLKDLWR

>WP_091898677.1 lactate oxidase [Marinilactibacillus piezotolerans]

MTNEEMIYNA PTEVKDIEVI NTYDLEEKVG KLLPKGGFGY IAGGVGDEYT LKQNIDAFNL KRILPRVLAD

VEHPETTTEV LGHELPSPII MAPIAAHGLA HESKEVGTAK GVSDYGTILS ISSYTGSDLY ETAKVVKGTS

KWFQLYMSKE NEVNKAVLDE AKADGATAII LTADATVMGN REKDLRNKFV FPFDMPIVSR HFRGSVEDEA

LNNIYLESKQ KINAEDIRFI ADYTKLPVLI KGVQSPEDAR LALDAGAAGI WVSNHGGRQL DGAPGAFEML

EEIAEVVDKR VPIVFDSGIR RGEHVFKALA SGADIVAIGR PVLYGLALGG WKGVRPVFNY FETDLKRVMQ

LAGTQTIEDV KRTELY

>WP_124974562.1 L-lactate oxidase [Ligilactobacillus salitolerans]

MAFKTSSAEG PVDFINTFDL EKMAQKVIPK GAFGYIASGA GDTFTLHENI RSFNHKLIVP HTLMDVEEPE

TKTIFAGEKL TAPMIMAPVA AHKLANEQGE TASAKGVADF GSIYTLSSYS SVDLPEVTAA LHGAPEWFQF

YFSKDNGINR HIMDRVKEQG IKAIVLTADA MVGGNRETDA RNNFTFPVTL PIVQEYLPDS VGKTMDYVYK

SAKQKLSPKD VEFIAEYSGL PVYVKGPQSK EDVDRALSAG AAGIWVSNHG GRQLDGGPAA FDSLQEVAQA

VDHRVPVIFD SGIRRGQHIF KAIASGADLV ALGRPVIYGL GLGGSVGVKQ VFEKLQAELK LVMQLAGTQT

VEDIKNFNLR DNHYDPAFDT SHDLKLV

>NBK09062.1 lactate oxidase [Enterococcus asini]

MEKIYQASTA EGAIDFINME DLETAAKEII PKGGYGYISS GAGDIFTYQE NERAFNHKLI IPHVLRDVEL

PDTTTKFGDE QLTAPIIMAP VAAHGLANIA AEKASAKGVA RFGTIYTASS YASCTLEEIR AAGGMEAPQW

FQFYMSKDDG INRDILEMAK RNGAKAIVLT ADATVGGNRE TDRRNGFTFP LAMPIVQAYQ SGIGQTMDAV

YGSSKQKLSP KDVEFIASHS ELPVYVKGVQ SEEDVERSLA SGASGIWVSN HGGRQLDGGP AAFDSLQYVA

EAVAGRAPIV FDSGVRRGQH VFKALASGAD LVAIGRPVIY GLALGGGTGV QQVFEFFKKE LEMVMQLAGT

QTITDIKQTR LRENGYL

>WP_231549421.1 alpha-hydroxy-acid oxidizing protein [Carnobacterium jeotgali]

MTETEKDVKI THSLDNIQEE STKANKIPYQ ASTAEKPLNI INVFDLELEA KKVIPEGGYG YISSGAGDLW

TIKQNIESFN HKLIVPRVLK NIEHPDQSTS IFGAELSTPI IMAPVASHGL ANVAAEPATA KAVAESGSIM

TISSYANKPF KEISQAGAGA PQWFQFYMSK DDGINRDILD EAKANGVKAI VLTADATVGG NREADKRNGF

VFPLGMPIVQ AYQSGVGQSM DAVYGSSKQT LSPKDVEFIA SYSGLPVFVK GVQTAEDALI SLASGAGGIW

VTNHGGRQLD GGPAAFDSLQ TVAEAVDRKV PIVFDSGVRR GQHVFKALAS GADLVAIGRP AIYGLALGGS

QGVKSVFDHF KHELELVMQL AGTKTVEDIK NTVLLDNRHA

>WP_057880708.1 lactate oxidase [Companilactobacillus kimchiensis]

MKKKYEASTA ENHVEIVNIA GLEARVKDHM SNEKGAFGYI RGGAEDEYTM KENTAAFNKA KIMPRVLQGI

DHADLSTNLW DIALKTPIIE SPSAAQGLAH ANGEKDTAKG VAAAGSIFSM STYGSTSLED GAAAAPDAPQ

FFQLYMSKDD KFNEFLINKA VKAGVKAIVL TVDSTLGGYR EEDVINKFQF PLPMPNLAAY SEGDGEGKGI

GEIYAAAKQG IVPSDIQKIK DMSGLPVIVK GIQSPDDADL AIEFGADGIW VSNHGGRQLD GAPASFDILP

DIADRVDKRV PVIFDSGVRR GEHVFKALAS GADLVAVGRP IIYGLNLGGA QGVTDVIEHL NMELSITMQL

AGTKTINDVK NTDLYY

>WP_195922460.1 lactate oxidase [Lactobacillus crispatus]

MTVYYKGFPQ SDRDEYLHMI NLQELEEKVK QVMPEGAYYY IASGAENEWT WRNNTAAFNH YQIVPRALTN

MQDPQTDTEF MGMKLKTPVM ISPIACHGIA HKDAEIATQK GAAAAGALFA SSTYANKSVE DIAAAAPNAP

RFFQLYLSKD WKFNRMVFDA IKKTGYKGIY LTVDALVSGY REANLRTHFT YPVPLDFFTR YMGAKGEGQS

VAQMYASSAQ KIGPKDVQRI KDETGLPVIV KGIECPEDAF KAIGAGADGV YVTNHGGREV DGGPATIDVL

PSIAKAVAHR VPIIFDSGVR RGSHVFKALA LGADMVGIGR PYLYGLALGG AKGVESVIDQ LDKELKIDMQ

LTGCKTIEDV KHAKIDRISY TADNLPSNTS PSRMKPYPVT ADNQIKKDTA ADASSGASEH

>WP_057824577.1 lactate oxidase [Lentilactobacillus sunkii]

MTVVNGYKQN DTEQKLDILN LPELEEKAKQ IIPTGGFGYI AGGSENNWTL KANRTAFTHK QIVPRALSNI

EKPELDTNVF GIPLKTPIMM APTAAQGLAH SQGEKDTAKG VAAVGGLMAQ STYSSTSIAD TAAAGNGAPQ

FFQLYMSKDW DFNYSLLDEA KKAGVKGIIL TVDATVDGYR EDDIKNNFQF PIPMANLTKF SEGDGKGKGI

GEIYASAAQK IGPDDVKKIA DYTDLPVIVK GIESPEDALY AIGAGAAGVY VSNHGGRQLN GGPASFDVLE

DVAKAVNGQV PIIFDSGVRR GSDVFKALAS GADLVAMGRP VIYGLALGGA EGVQSVFEHL GDELKIIMQL

AGTKTIADVK KANLLNIKY

>XP_020776044.1 hydroxyacid oxidase 2 [Boleophthalmus pectinirostris]

MSMVCLTDFE EYAKEHLSKA TWDYYAAGAD ECCTRDDNLL AYKRIRLRPR ILRDVSVCDT RTTVQGTEIS

FPVGIAPTAF HCLAWHEGEV ATARATEALN TCYITSTYST CSVEEIVAAA PNGYRWFQLY VYRDRKLSEH

IVQRAESLGY KALVLTVDVP YTGKRRNDIR NQFKLPPHLK VKNFDGVFQQ AEAYGVPPDT LDPSISWKDV

QWLQSITRLP IIIKGILTKE DAELAVEHGV QGIIVSNHGG RQLDGGPASI DALAEIVNTV RGRIEVYVDG

GIRTGSDVLK ALALGAKCVF IGRPAIWGLA YKGEEGVREV LQILNDEFRL SMALSGCRNV AEISRDLIQC

SKL

>XP_040607440.1 hydroxyacid oxidase 2 [Mesocricetus auratus]

MSLVSLADFK AQAQEHLSKS SWDFIEGEAD EGITYKDNLA AFKRIRLRPR YLRDVSEIDT RTIIQGQEIE

APICISPTAF HSIAWPDGER STARAAQEAN ICYITSTYAS CTLEDIVAAA PRGFRWFQLY VQSDWELNKQ

LIQRVEGLGF KALVITVDAP VIGKRRRNIK NQLNLEANIM LKDLRSPEAR NSTHFSPMSL PSASFCWNDL

SLLQSITRLP IILKGILTKE DAELAVKHHV QGIFVSNHGG RQIDEVPASI DALMEVVAAV KGKVEVYMDG

GVRTGNDVLK ALALGAKCIF LGRPILWGLA CKGEDGVKEV LSILKGEFQT SMALSGCRSI AEISPDLIQF

SRL
